# An Essay on the Treatment of Compound and Complicated Fractures, Being an Annual Address before the Massachusetts Medical Society, in Boston, U. S. America, May 28, 1845

**Published:** 1847-01

**Authors:** 


ART. XXIV.—*An Essay on the Treatment of Compound and Complicated Fractures, being an Annual Address before the Massachusetts Medical Society, in Boston, U. S. America, May* 28, 1845. By WILLIAM J. WALKER, M.D., Fellow of the Society—*Boston,* 1845. 8vo, pp. 100.

WE have no personal knowledge of the author of this pamphlet, but should suppose, from internal evidence, that he is an easy old gentleman who at some trouble had delivered himself of an oration, and being mightily pleased with his own prowess as displayed thereby, had resolved to perpetuate the event in print. The work, however, although containing nothing striking or important, is creditable enough.

It consists of an introductory section, and sundry cases of extensive injury, culled mainly from the older authors. The object is stated to be the elucidation of certain principles in practice, which—so far as we understand them—have been, for many years, on this side of the water, regarded as fixed and determined. The young surgeon, however, may profit by their re-statement:

"These cases, as I think, prove the powers of Nature in healing compound fractures attended with great contusion, laceration, and loss of substance of the bones, and of the skin, and other soft parts, to be much greater than is generally supposed. They also show us that large portions of bone, when lost by accident, or removed by art, may be reproduced by the powers of Nature; and they illustrate, in a striking manner, the truth and importance of certain practical precepts established by the older writers on surgery, with respect to the treatment of wounds, complicated with fractures of the bones. The precepts to which I allude are:

"First, that all tendinous or membranous structures which obstruct the removal of foreign bodies, or unduly confine or strangulate the soft parts, when swollen by inflammation, should be promptly and freely divided.

"Second, that such dependent orifices should be preserved, or counter-openings made, as will, when aided by position or dressings, secure the free discharge of all fluids which might otherwise stagnate within the wound.

"Third, that portions of bone protruding through the integuments which cannot easily and without violence be reduced to their proper place, should at once be removed by the saw; and that all foreign bodies, loose portions, or shivers of bone, should be promptly extracted from the wound.

"Fourth, that great pain, inflammation, or nervous symptoms depend rather on peculiar complication, than on the exent of the wound. And that they indicate great danger unless rightly understood, appropriately treated, and relieved." (pp. 3-5.)

The only objection we have to his doctrines is, that in illustrating the last clause of his fourth head, the author rather runs into an extreme; advocating the incision of tendinous and membranous structures at once; not waiting to see whether or not, or to what extent, they may be implicated injuriously. In fact, he would seek to revive a rule of practice in regard to fractures, similar to the exploded treatment of punctured wounds; which wont to be dilated on the spot, simply because they came under the special category of "punctured." There is nothing worse in surgery than a wanton and uncalled-for use of the knife; nothing to be more studiously deprecated and shunned.

